# STRESS, DEPRESSION, AND COGNITIVE FUNCTION IN ADRD FAMILY CAREGIVERS

**DOI:** 10.1093/geroni/igz038.1810

**Published:** 2019-11-08

**Authors:** Frank Puga, Kylie Meyer, Carolyn E Pickering

**Affiliations:** 1 University of Texas Health San Antonio, San Antonio, Texas, United States; 2 University of Texas Health Science Center San Antonio, San Antonio, Texas, United States

## Abstract

The rapidly growing number of individuals over the age of 55 stresses the need to identify unique factors to decrease older adults’ vulnerability to psychiatric and neurodegenerative disorders, especially among high-risk groups such as family caregivers to persons with Alzheimer’s disease or related dementias (ADRD). In this project, we investigated physiological stress responses, depression, and cognitive functioning. Using data from the Midlife in the United States database, we examined differences in physiological stress responses, depressive symptoms, and cognitive function (including working memory, episodic memory, verbal ability, processing speed, and executive functioning) between ADRD and non-ADRD caregivers. Results from the secondary analysis revealed a decrease in cognitive function, an increase in depressive symptoms and disrupted physiological responses in ADRD caregivers. These data suggest that the unique aspects of ADRD caregiving are a risk factor for the onset of psychiatric and neurodegenerative disorders. The results of this study support the development of targeted interventions to reduce the risk of depression and cognitive decline among ADRD caregivers. The specific focus on older adult ADRD caregivers, physiological processes, mental health, and dementia is unique in the field of aging and provides a critically needed new paradigm for identifying strategies to support ADRD caregivers and understanding the development of ADRD.

